# Anterior Cruciate Ligament‐Return to Sport after Injury (ACL‐RSI) subcategories are affected by subjective running ability and medial single‐leg hop distance in postreconstruction patients at 6 months

**DOI:** 10.1002/jeo2.12004

**Published:** 2024-02-03

**Authors:** Jun‐ya Aizawa, Kenji Hirohata, Shunsuke Ohji, Sho Mitomo, Takehiro Ohmi, Hideyuki Koga, Kazuyoshi Yagishita

**Affiliations:** ^1^ Department of Physical Therapy Juntendo University Tokyo Japan; ^2^ Department of Rehabilitation Medicine, Graduate School of Medical and Dental Sciences Tokyo Medical and Dental University Tokyo Japan; ^3^ Clinical Center for Sports Medicine and Sports Dentistry Tokyo Medical and Dental University Tokyo Japan; ^4^ Japan Sports Agency Tokyo Japan; ^5^ Department of Joint Surgery and Sports Medicine, Graduate School of Medical and Dental Sciences Tokyo Medical and Dental University Tokyo Japan

**Keywords:** ACL‐RSI, confidence, emotions, physical function, risk appraisal

## Abstract

**Purpose:**

This study aimed to investigate the intricate relationship between physical function factors and each subcategory score of the Anterior Cruciate Ligament‐Return to Sport after Injury (ACL‐RSI) scale among patients following ACL reconstruction.

**Methods:**

Participants comprised 59 patients who had undergone primary ACL reconstruction using hamstring tendon. The ACL‐RSI was completed 6 months after reconstruction and five physical functions were measured in patients on the same day. Simple linear regression was performed multiple times to investigate the relationship between ACL‐RSI subcategory scores as a dependent variable and each independent variable (knee strength, leg anterior reach distance, single‐leg hop [SLH] distances, side bridge endurance, and subjective running ability). Multiple regression analysis was performed using a stepwise method, with factors showing a risk rate <0.05 in simple linear regression analyses as independent variables and the ACL‐RSI in each subcategory score as the dependent variable.

**Results:**

Multiple regression analysis showed that subjective running ability affected all subcategories (*p* ≤ 0.001), and that the limb symmetry index of medial SLH distance affected both the Emotions (*p* = 0.047) and Confidence (*p* = 0.009) subcategories. Higher subjective running ability and greater limb symmetry in the medial SLH were thus positively associated with each dimension of psychological readiness.

**Conclusions:**

This study highlights the differential impact of physical function factors on specific subcategories of the ACL‐RSI scale, providing clinicians with insights for designing targeted rehabilitation strategies. This original paper suggests the importance of analysing factors related to subcategory scores in addition to total ACL‐RSI score, and could contribute to the understanding of determinants for a successful return to sport following ACL reconstruction.

**Level of Evidence:**

Level IV.

**Abbreviations**
ACL: anterior cruciate ligament
ACL‐RSI: Anterior Cruciate Ligament‐Return to Sport after Injury scale
height ratio: ratio of the distance measured to the height of the patient
HQ ratio: hamstring‐to‐quadriceps ratio
ICC: intraclass correlation coefficient
LSI: limb symmetry index
SLH: single‐leg hop
weight ratio: ratio of peak torque measured to body mass of the patient

## BACKGROUND

Many patients who sustain damage to the anterior cruciate ligament (ACL) undergo reconstruction. After ACL reconstruction, these individuals require long‐term rehabilitation to improve physical function and return to the sport they were engaged in before injury [1]. However, only 44%–63% of patients are able to return to their chosen sport [2], and around 17% of elite athletes do not end up returning to their sport [1].

A combination of emotions, confidence, and risk appraisal represent psychological readiness, contributing to the ability to return to sport after reconstruction [3, 4]. The Anterior Cruciate Ligament‐Return to Sport after Injury (ACL‐RSI) scale is a 12‐item scale developed to quantify the state of psychological readiness during recovery after injury and reconstruction [4, 5]. The ACL‐RSI score of a patient after reconstruction has been shown to correlate with whether the individual will return to their sport [4, 6, 7, 8]. Lower ACL‐RSI total score has been associated with the occurrence of secondary injuries after reconstruction [9]. On the other hand, the Risk Appraisal subcategory score of ACL‐RSI at 7 months after reconstruction has been reported to be higher for those who sustained a second injury within 2 years after surgery than for those who did not experience reinjury [10]. In recent years, inclusion of the ACL‐RSI score in criteria for the return to sport has been recommended [3, 7]. Information about specific factors that contribute to psychological readiness is therefore needed to plan the rehabilitation required to achieve a return to sports.

An improved state of psychological readiness at 6 months after reconstruction has been reported as important for returning to sports at preinjury levels [7]. The degree of improvement in ACL‐RSI subcategory scores differs from 1 day before reconstruction to 6 months after surgery, with these differences reflected in the ACL‐RSI total score [11]. There is one study that analysed the relationship between subcategory score and physical function variable, but this study only analysed the association between Risk Appraisal score and laterality in anterior single‐leg hop (SLH) distance among patients who had not returned to sports at 2 years postoperatively [12]. Six months after reconstructive surgery is considered an appropriate time to begin sports‐specific training in earnest, with the aim of improving long‐term outcomes and returning to play. Previous cross‐sectional studies have revealed that at 6 months after reconstruction, increased knee strength and hamstring‐to‐quadriceps ratio at different angular velocity, leg anterior reach distance symmetry, lateral and medial SLH distances, and subjective running ability appear important to exceed the threshold ACL‐RSI total score allowing a return to sports [13]. While a previous study of patients at 6 months after reconstruction reported physical function factors associated with the ACL‐RSI total score [13, 14], no previous studies have reported factors associated with subcategory scores at 6 months after reconstruction.

The study aimed to investigate the intricate relationship between physical function factors and each subcategory score of the ACL‐RSI scale among patients following ACL reconstruction. We hypothesized that subjective running ability, lateral and medial SLH distances, and knee strength would represent significant contributors to various ACL‐RSI subcategories.

## METHODS

### Participants

**Figure 1:**
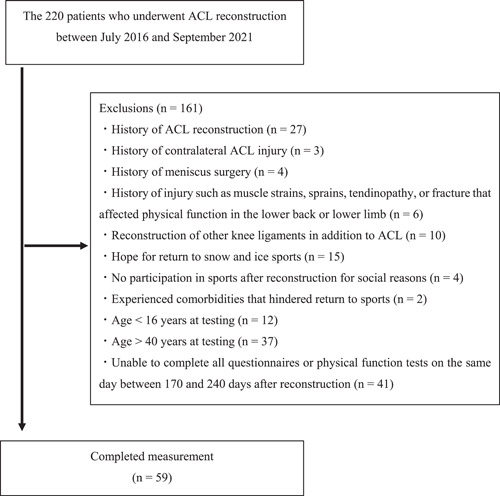
Flowchart of patient inclusion in the study. ACL, anterior cruciate ligament.

Patients in this cross‐sectional study were selected from a list of the name, age, sex, and date of surgery for all 220 patients who underwent ACL reconstruction between the start of July 2016 and the end of September 2021 in the Department of Joint Surgery and Sports Medicine at a single center (Figure 1). ACL‐RSI and physical functions were measured at 6 months after reconstruction. Inclusion criteria were: primary/unilateral anatomical double‐bundle reconstruction using either hamstring tendon autograft alone or gracilis tendon harvested in addition to hamstring tendon or patellar tendon autograft; age ≥16 years but ≤40 years at the time of testing; postoperative rehabilitation under the same protocol used in the Department of Sports Physical Therapy; and participation in training sessions for the same sport the patient had been participating in before ACL injury at approximately 6 months after reconstruction [14].

Patients were excluded if they had: a history of ACL reconstruction; history of contralateral ACL injury; history of meniscus surgery; history of injuries such as muscle strains, sprains, tendinopathy, or fracture that affected physical function in the lower back or lower limb after reconstruction or within the 6 months preceding reconstruction; history of reconstruction for other knee ligaments in addition to the ACL; hope to return to snow and ice sports such as skiing or ice hockey; no participation in sports after reconstruction for social reasons such as relocating or becoming pregnant; experience of comorbidities that hindered the return to sports; or inability to complete the ACL‐RSI and physical function tests on the same day between 170 and 240 days after reconstruction [14]. Patients wishing to return to snow and ice sports were excluded because these sports involve distinctly different surfaces, shoes, and injury mechanisms such as slip‐catch while skiing and involvement of centrifugal force during figure skating [15, 16].

### Postoperative rehabilitation

The protocol for postoperative rehabilitation was the same for all patients [17]. However, patients who underwent repair of the middle posterior segment of the meniscus were prohibited from deep squatting until 3 months after surgery [18, 19]. Thirty‐seven patients underwent repair of the middle posterior segment of the meniscus (lateral meniscus only, *n* = 25; medial meniscus only, *n* = 7; both, *n* = 5). Patients were permitted to begin isometric quadriceps exercises as tolerated from the day after reconstruction. Using a knee brace (Straighten Position Knee‐Joint Immobilizer; ALCARE) and crutches, partial weight‐bearing (20 kg) was permitted on the first day after reconstruction, gradually increasing to full body weight‐bearing for each patient. Use of the knee brace and crutches was discontinued at 4 weeks after reconstruction. Range‐of‐motion exercises from full extension to 120° of flexion were started on the postoperative Day 2. Closed kinetic chain exercises such as weight shifting and squatting were started –2 weeks after reconstruction. A heel slide exercise was started 3 days after reconstruction. This is an exercise in which the patient bends the knee while sliding the heel on the bed in a long sitting position [20]. Two weeks after reconstruction, a curl exercise was started to actively bend the knee in a prone position [21]. A hip‐lift exercise to raise the buttocks in the crook lying position was started 4 weeks after reconstruction. Patients were instructed to refrain from repeated knee extension training with maximum resistance near the ankle in a sitting position within the range of 10–30° of knee flexion for 3 months after reconstruction [22]. All exercises were performed while confirming that no pain was present in the area from which the tendon was harvested.

Running exercises were started in athletes who had cleared the criterion of limb symmetry index (LSI) ≥65% for knee isokinetic extension strength as measured by the Biodex Multi‐Joint Testing and Rehabilitation System (BDX‐4; Biodex Medical Systems) at 3 months after reconstruction. Speed and distance of running were gradually increased for joint effusion and symptoms of each patient. Once 80% of subjective full‐speed running ability had been achieved, exercises related to the desired sporting activities were initiated with detailed instructions. All exercises were specific to the individual patient, depending on the type of sport and position played.

Participation in sports exercises with limited contact was allowed from 6 months after reconstruction, as long as the patient showed no problematic symptoms in the joint and displayed sufficient knee isokinetic flexion/extension strength (LSI > 80%) and SLH distance (LSI > 80%) after the specified training without contact had been completed [17]. Criteria for determining when to return to participation in the actual sport were: ≥8 months after reconstruction [14]; LSI of flexion/extension strength ≥90% [23]; LSI of SLH distance ≥90% [23]; ACL‐RSI score ≥60 [5, 7]; and subjective running ability ≥90% [14].

### Study protocol

Physical functions were measured on the same day the ACL‐RSI was completed. Rest intervals of 10 min were provided between knee strength tests, the leg anterior reach test, SLH tests, and the side bridge endurance test. All physical functions were measured by five physiotherapists (J. A., K. H., S. O., T. O., and S. M.), each with more than 10 years of clinical experience in rehabilitation for patients after ACL reconstruction.

### Participant characteristics

Sex was determined based on medical records. Height and body mass were measured on the same testing day, and body mass index was calculated. The level of sports activity before injury was graded using the modified Tegner activity scale [24]. Participants were interviewed regarding the mean time (in hours) spent participating in sports the week before injury. Sports participated in before injury were classified into four types according to the criteria described by Montalvo [25]: collision; contact; limited contact; and noncontact. Injury situations were elicited from participants and classified into three categories: noncontact; indirect contact; and direct contact [26]. Date of injury and date of reconstruction were confirmed both by the participant and from medical records, then the interval (in days) from date of injury to date of reconstruction was calculated. The number of days after reconstruction was recorded as the number of days from reconstruction to testing.

Types of autograft (semitendinosus tendon, semitendinosus plus gracilis tendon, and patellar tendon) were confirmed from detailed records made during reconstruction. Meniscus injuries and treatments were confirmed from detailed records of arthroscopic findings during reconstruction. The injured segment (anterior, middle, or posterior), injury type (longitudinal, radial, or horizontal), and treatment method (suture, centralization, or partial meniscectomy) were confirmed. Participants were defined as being treated regardless of the method used. Lateral extra‐articular tenodesis was confirmed from surgical records.

### Dependent variable: Psychological readiness to return to sport

*ACL‐RSI*. Patients completed the ACL‐RSI, which includes three subcategories: Emotions, Confidence, and Risk Appraisal. Scores for each subcategory are summed and averaged, providing a total score between 0 and 100. Higher scores indicate greater psychological readiness. This scale has been validated and its predictive value has been demonstrated in previous studies [6, 27]. The Japanese version of the ACL‐RSI has been confirmed to offer a highly practical questionnaire with good face validity and internal consistency [5]. The scale includes five questions on Emotion, five on Confidence regarding physical performance, and two on Risk Appraisal. Each question is scored from 0 to 100, with 0 representing the worst outcome and 100 the best. In addition to total score, we estimated each subcategory of the ACL‐RSI scale, with each perfect score calculated as 100 points. Because the Emotion and Confidence subcategories each had five questions, total score was calculated after dividing those subcategory scores by five. Because the Risk Appraisal subcategory had two questions, total score was calculated after dividing that subcategory score by two.

### Independent variables: Physical function scores

The Biodex Multi‐Joint Testing and Rehabilitation System torque metre was used to evaluate isokinetic strength of the knee in extension/flexion as described in previous research [13, 14]. Results are presented as the ratio of peak torque measured to body mass of the patient (weight ratio) and LSI. Hamstring‐to‐quadriceps ratio (HQ ratio) was calculated as the ratio of peak torque for the hamstring to peak torque for the quadriceps. The test–retest reliability of concentric peak torque for the knee using the Biodex System has been reported as “high” to “very high” [28]. Leg anterior reach distance with maximal effort was measured using a Y Balance Test Kit (Functional Movement Systems®) according to previous research [13, 14, 29]. Results are represented as the ratio of reach distance to lower limb length of the patient and LSI.

SLH distances in the three directions (anterior, lateral, and medial) were measured in random order, in accordance with previously reported protocols [13, 14]. Results are represented as the ratio of the distance measured to patient height (height ratio) and LSI. Intraclass correlation coefficient (ICC) was calculated to examine the reproducibility of SLH distances in the three directions for the involved and uninvolved limbs of 10 athletes who met the same inclusion criteria applied in this study. To determine ICCs, SLH distance was measured three times in a single day and ICCs of 1–3 measured values were calculated in each direction. As a result, the ICCs of single measurement values of the involved and uninvolved limbs were within the ranges of 0.91–0.99 and 0.91–0.96, respectively, showing “almost perfect” reproducibility [30]. The side bridge endurance test was measured using the method described by McGill et al. [31]. The side bridge endurance test has been found to show high intra‐ and interrater reliability [32]. Side bridge endurance was included as an independent variable based on systematic reviews that demonstrated that poor core stability may contribute to an increased risk of ACL injury in young athletes [33]. Participants were asked about subjective running ability, with maximal effort representing 100% of ability before injury, using the following question: “What do you think your current straight running ability is now, if your straight running ability at full power before the injury was 100%?” [13, 14].

### Statistical analyses

The normality of each variable was confirmed using the Shapiro–Wilk test. Normally distributed data for continuous variables are summarized as means and standard deviations. Nonnormally distributed data are summarized as medians and interquartile ranges. Simple linear regression was performed multiple times to investigate the relationship between ACL‐RSI subcategory scores as a dependent variable and each independent variable (knee strength, leg anterior reach distance, SLH, side bridge endurance, and subjective running ability). Multiple regression analysis was performed using a stepwise method, with factors showing a risk rate <0.05 in simple linear regression analyses as independent variables and the ACL‐RSI in each subcategory score as the dependent variable. All data were analysed using the Statistical Package for the Social Sciences for Windows (version 21.0; IBM Corp.). Values of *p* < 0.05 were considered indicative of statistical significance. Post hoc power analysis was performed using the G*Power software package (version 3.1.9.7; Kiel University). For Emotions, Confidence, and Risk Appraisal, post hoc power analysis of the main result (multiple regression) showed effect sizes of 0.254, 0.416, and 0.165, corresponding to power (1 – *β*) of 93.1%, 99.4%, and 86.6%, respectively.

## RESULTS

### Participants characteristics (Table 1)

**Table 1:** Participants characteristics (*N* = 59).

Age, years	20.0 (6.0) [20.4–23.2]
Women:men	35:24
Height, cm	166.3 ± 8.6 [164.1–168.5]
Body mass, kg	61.0 (19.0) [60.5–66.8]
Body mass index, kg/m^2^	22.9 ± 2.9 [21.1–23.6]
ACL‐RSI score (0–100)	63.8 ± 17.9 [59.1–68.4]
Emotions (0–100)	59.9 ± 21.1 [54.4–65.4]
Confidence in performance (0–100)	65.9 ± 19.8 [60.7–71.0]
Risk Appraisal (0–100)	68.1 ± 18.7 [63.2–72.9]
Preinjury modified Tegner activity scale score	8.0 (2.0) [6–10]
Preinjury sports participation time, h/week	6.0 (8.0) [6.7–10.0]
Participating sport type (collision; contact; limited contact; noncontact)	6; 38; 7; 8
Injury situations (noncontact; indirect contact; direct contact)	37; 15; 7
Days from injury to reconstruction	67.0 (70.0) [69.4–145.2]
Meniscus treated:nontreated, *n*	40:19
Autograft (ST; STG; PT)	48; 4; 7
Days from reconstruction to testing	187.0 (14.0) [184.7–191.8]

*Note*: Data are reported as mean ± SD or median (interquartile range) [95% confidence interval] unless otherwise indicated. For preinjury modified Tegner activity scale score, data are reported as median (interquartile range) [minimum and maximum values].

Abbreviations: ACL‐RSI, Anterior Cruciate Ligament‐Return to Sport after Injury Scale; PT, patellar tendon; ST, semitendinosus; STG, gracilis tendon in addition to semitendinosus.

Fifty‐nine participants were included in the study. The median age of the participants was 20 years, and 59% were women. Median preinjury Tegner score was 8.0 and mean interval from reconstruction to testing was 187.0 days. No patients underwent lateral extra‐articular tenodesis.

### ACL‐RSI scores (Table 1)

Mean ACL‐RSI total score was 63.8. Mean ACL‐RSI subcategory scores for Emotions, Confidence, and Risk Appraisal were 59.9, 65.9, and 68.1, respectively.

### Physical function scores (Table 2)

**Table 2:** Physical function scores (*N* = 59).

	Involved limb, N m/kg	Uninvolved limb, N m/kg	LSI, %
Knee extension strength (60°/s)	2.06 ± 0.38 [1.96–2.16]	2.52 ± 0.46 [2.40–2.64]	82.52 ± 11.42 [79.55–85.50]
Knee flexion strength (60°/s)	1.09 ± 0.25 [1.03–1.16]	1.25 (0.36) [1.19–1.40]	87.20 ± 16.23 [82.97–91.43]
Knee extension strength (180°/s)	1.45 ± 0.25 [1.38–1.51]	1.79 ± 0.31 [1.71–1.87]	81.06 ± 9.11 [78.69–83.44]
Knee flexion strength (180°/s)	0.85 ± 0.21 [0.79–0.90]	0.97 ± 0.20 [0.92–1.02]	87.51 ± 13.32 [84.04–90.98]

	Involved limb, %	Uninvolved limb, %	
HQ ratio (60°/s)	53.23 ± 9.37 [50.78–55.67]	49.25 (10.25) [48.00–54.12]	
HQ ratio (180°/s)	58.56 ± 9.99 [55.96–61.17]	54.26 ± 7.73 [52.24–56.27]	

	Involved limb, % lower limb length	Uninvolved limb, % lower limb length	LSI, %
Leg anterior reach distance	69.6 ± 6.3 [68.0–71.3]	71.9 ± 6.5 [70.2–73.6]	97.0 ± 4.9 [95.7–98.3]

	Involved limb, % height	Uninvolved limb, % height	LSI, %
Anterior SLH distance	62.1 ± 14.8 [58.2–66.0]	71.9 ± 14.5 [68.1–75.7]	89.5 (16.4) [83.1–89.9]
Lateral SLH distance	47.4 ± 12.5 [44.2–50.7]	56.2 ± 11.7 [53.2–59.2]	84.1 ± 13.6 [80.5–87.6]
Medial SLH distance	52.1 ± 13.4 [48.7–55.6]	58.5 ± 12.6 [55.2–61.8]	88.5 (16.0) [85.5–92.7]

	Involved side, sec	Uninvolved side, sec	LSI, %
Side bridge endurance	62.0 (28.0) [57.5–71.2]	67.0 (31.0) [58.0–71.3]	98.4 (21.8) [95.0–105.1]
Subjective running ability, %	80.0 (28.0) [78.2–86.4]		

*Note*: Data are reported as mean ± SD or median (interquartile range) [95% confidence interval].

Abbreviations: HQ ratio, hamstring‐to‐quadriceps ratio; LSI, limb symmetry index; SLH, single‐leg hop.

Mean LSI for knee extension strength (180°/s) was 81.1%. Mean LSIs for anterior and lateral SLH distances were 89.5% and 84.1%, respectively. Median subjective assessment of running ability was 80.0% of preinjury ability.

### Simple regression analyses between physical function scores and ACL‐RSI subcategory scores (Table 3)

**Table 3:** Simple linear regression to identify physical function factors associated with ACL‐RSI subcategory score (*N* = 59).

	Total	Emotions	Confidence	Risk appraisal
Knee extension strength (60°/s)				
Involved limb, N m/kg	**0.280 [1.195 to 25.081]** * **0.032**	0.222 [−2.016 to 26.600] 0.091	**0.287 [1.696 to 28.032]** * **0.028**	0.224 [−1.680 to 23.719] 0.088
Uninvolved limb, N m/kg	0.147 [−4.484 to 15.926] 0.226	0.120 [−6.583 to 17.582] 0.366	0.157 [−4.498 to 18.017] 0.234	0.091 [−7.053 to 14.471] 0.493
LSI, %	0.152 [−0.173 to 0.648] 0.251	0.113 [−0.277 to 0.696] 0.393	0.144 [−0.205 to 0.703] 0.277	0.173 [−0.145 to 0.712] 0.190
Knee flexion strength (60°/s)				
Involved limb, N m/kg	0.152 [−7.941 to 29.930] 0.250	0.082 [−15.567 to 29.480] 0.539	0.212 [−3.789 to 37.587] 0.107	0.084 [−13.636 to 26.357] 0.527
Uninvolved limb, N m/kg	0.101 [−7.136 to 15.992] 0.446	0.052 [−11.019 to 16.365] 0.679	0.135 [−6.200 to 19.250] 0.309	0.079 [−8.516 to 15.757] 0.553
LSI, %	−0.043 [−0.339 to 0.244] 0.746	−0.056 [−0.417 to 0.271] 0.671	−0.025 [−0.353 to 0.292] 0.851	−0.022 [−0.332 to 0.280] 0.866
HQ ratio (60°/s)				
Involved limb, %	−0.048 [−0.596 to 0.415] 0.721	−0.067 [−0.746 to 0.446] 0.616	0.007 [−0.545 to 0.574] 0.958	−0.104 [−0.735 to 0.320] 0.433
Uninvolved limb, %	0.058 [−0.314 to 0.492] 0.660	0.019 [−0.442 to 0.511] 0.884	0.091 [−0.292 to 0.597] 0.495	0.042 [−0.355 to 0.491] 0.749
Knee extension strength (180°/s)				
Involved limb, N m/kg	**0.265 [0.671 to 36.810]** * **0.042**	0.168 [−7.766 to 35.816] 0.203	**0.313 [4.792 to 44.121]** * **0.016**	0.221 [−2.810 to 35.482] 0.093
Uninvolved limb, N m/kg	0.081 [−10.662 to 20.112] 0.541	0.055 [−14.402 to 21.963] 0.679	0.113 [−9.666 to 24.229] 0.393	0.012 [−15.452 to 16.887] 0.930
LSI, %	**0.286 [0.063 to 1.060]** * **0.028**	0.184 [−0.178 to 1.028] 0.164	**0.307 [0.119 to 1.213]** * **0.018**	**0.313 [0.126 to 1.161]** * **0.016**
Knee flexion strength (180°/s)				
Involved limb, N m/kg	0.221 [−3.178 to 40.308] 0.093	0.172 [−8.835 to 42.973] 0.192	0.254 [−0.194 to 47.448] 0.052	0.110 [−13.499 to 32.919] 0.406
Uninvolved limb, N m/kg	0.167 [−8.325 to 37.720] 0.206	0.168 [−9.745 to 44.562] 0.204	0.171 [−8.744 to 42.091] 0.194	0.033 [−21.378 to 27.512] 0.803
LSI, %	0.149 [−0.152 to 0.552] 0.259	0.091 [−0.274 to 0.562] 0.492	0.184 [−0.113 to 0.660] 0.162	0.112 [−0.213 to 0.528] 0.399
HQ ratio (180°/s)				
Involved limb, %	0.083 [−0.325 to 0.621] 0.533	0.126 [−0.289 to 0.821] 0.342	0.069 [−0.387 to 0.660] 0.604	−0.063 [−0.615 to 0.378] 0.634
Uninvolved limb, %	0.148 [−0.264 to 0.949] 0.263	0.188 [−0.198 to 1.224] 0.154	0.110 [−0.392 to 0.955] 0.406	0.029 [−0.572 to 0.713] 0.826
Leg anterior reach distance				
Involved limb, % lower limb length	0.104 [−0.453 to 1.042] 0.433	0.046 [−0.731 to 1.041] 0.728	0.147 [−0.360 to 1.283] 0.266	0.076 [−0.558 to 1.012] 0.565
Uninvolved limb, % lower limb length	0.059 [−0.569 to 0.896] 0.657	0.054 [−0.687 to 1.042] 0.682	0.027 [−0.729 to 0.892] 0.841	0.115 [−0.431 to 1.096] 0.387
LSI, %	0.077 [−0.680 to 1.237] 0.563	−0.017 [−1.207 to 1.061] 0.897	0.212 [−0.187 to 1.889] 0.106	−0.074 [−1.285 to 0.724] 0.579
Anterior SLH distance				
Involved limb, % height	**0.261 [0.006 to 0.623]** * **0.046**	0.176 [−0.121 to 0.622] 0.182	**0.308 [0.075 to 0.747]** * **0.017**	0.184 [−0.098 to 0.561] 0.164
Uninvolved limb, % height	0.082 [−0.225 to 0.427] 0.537	−0.007 [−0.395 to 0.376] 0.961	0.168 [−0.128 to 0.584] 0.204	0.045 [−0.284 to 0.400] 0.735
LSI, %	**0.342 [0.126 to 0.805]** ** **0.008**	**0.313 [0.098 to 0.908]** * **0.016**	**0.310 [0.087 to 0.846]** * **0.017**	**0.259 [0.003 to 0.735]** * **0.048**
Lateral SLH distance				
Involved limb, % height	**0.261 [0.007 to 0.741]** * **0.046**	0.118 [−0.245 to 0.646] 0.372	**0.349 [0.160 to 0.947]** ** **0.007**	0.240 [−0.026 to 0.747] 0.067
Uninvolved limb, % height	0.076 [−0.289 to 0.522] 0.567	−0.050 [−0.569 to 0.389] 0.708	0.184 [−0.130 to 0.753] 0.163	0.090 [−0.279 to 0.569] 0.497
LSI, %	**0.373 [0.168 to 0.816]** ** **0.004**	**0.297 [0.068 to 0.855]** * **0.022**	**0.375 [0.187 to 0.903]** ** **0.005**	**0.315 [0.087 to 0.781]** * **0.015**
Medial SLH distance				
Involved limb, % height	**0.322 [0.095 to 0.767]** * **0.013**	0.236 [−0.035 to 0.779] 0.072	**0.371 [0.185 to 0.913]** ** **0.004**	0.203 [−0.080 to 0.648] 0.124
Uninvolved limb, % height	0.151 [−0.158 to 0.586] 0.254	0.079 [−0.310 to 0.575] 0.551	0.204 [−0.088 to 0.727] 0.122	0.105 [−0.237 to 0.548] 0.431
LSI, %	**0.347 [0.127 to 0.773]** ** **0.007**	**0.298 [0.068 to 0.843]** * **0.22**	**0.357 [0.157 to 0.868]** ** **0.05**	0.203 [−0.077 to 0.629] 0.123
Side bridge endurance				
Involved side, sec	0.127 [−0.093 to 0.266] 0.338	0.057 [−0.168 to 0.259] 0.670	0.197 [−0.048 to 0.344] 0.135	0.050 [−0.154 to 0.225] 0.708
Uninvolved side, sec	0.074 [−0.133 to 0.237] 0.576	−0.027 [−0.241 to 0.197] 0.841	0.159 [−0.080 to 0.325] 0.231	0.085 [−0.132 to 0.256] 0.523
LSI, %	0.144 [−0.109 to 0.376] 0.276	0.248 [−0.010 to 0.551] 0.059	0.075 [−0.193 to 0.347] 0.571	−0.071 [−0.325 to 0.187] 0.592
Subjective running ability, %	**0.558 [0.384 to 0.883]** ** **<0.00**	**0.446 [0.279 to 0.914]** ** <**0.00**	**0.584 [0.462 to 1.002]** ** <**0.00**	**0.406 [0.195 to 0.771]** ** <**0.00**

*Note*: Values represent simple linear regression standardized coefficients [95% confidence interval] *p* value.

Abbreviations: ACL‐RSI, Anterior Cruciate Ligament‐Return to Sport after Injury Scale; HQ ratio, hamstring‐to‐quadriceps ratio; LSI, limb symmetry index; SLH, single‐leg hop.

* Significance at *p* < 0.05.

** Significance at *p* < 0.01.

Among the subcategories, those physical functions that showed significant associations with only the Confidence subcategory were knee extension strength (60°/s, 180°/s) of the involved limb and SLH distances in the three directions of the involved limb. The LSI of knee extension strength (180°/s) was significantly associated with Risk Appraisal and Confidence subcategories. The LSI of medial SLH distance was significantly associated with Confidence and Emotions subcategories. The physical function variables associated with the three subcategories were subjective running ability and LSIs of lateral and anterior SLH distances.

### Multivariate regression to identify factors associated with ACL‐RSI subcategory score (Table 4)

**Table 4:** Multivariate regression to identify factors associated with ACL‐RSI subcategory score (*N* = 59).

Independent variables	*β* coefficient (95% CI)	*p* Value
Emotions		
Subjective running ability	0.411 (0.237–0.863)	0.001
Medial SLH distance LSI	0.421 (0.063–0.778)	0.047
Confidence		
Subjective running ability	0.543 (0.421–0.940)	<0.000
Medial SLH distance LSI	0.277 (0.101–0.694)	0.009
Risk Appraisal		
Subjective running ability	0.406 (0.195–0.771)	0.001

Abbreviations: ACL‐RSI, Anterior Cruciate Ligament‐Return to Sport after Injury Scale; LSI, Limb Symmetry Index; SLH, single‐leg hop.

For Emotions and Confidence, variables with significant partial correlations were subjective running ability (*p* = 0.001 and <0.000) and LSI of medial SLH distance (*p* = 0.047 and 0.009). For Risk Appraisal, subjective running ability showed a significant partial correlation (*p* = 0.001). The variance inflation factor did not exceed 10 for any variable, and no multicollinearity was apparent between variables.

## DISCUSSION

The study reports insights into the relationships between physical functions and ACL‐RSI scale subcategories. Notably, physical factors such as subjective running ability, SLH distances, and knee strength emerged as contributors to various ACL‐RSI subcategories, underlining their importance in specific dimensions of the return‐to‐sport process. The study highlights the association between SLH distances in multiple directions on the involved limb and the Emotions and Confidence subcategories. Subjective running ability was found to affect all subcategories. The LSI of medial SLH distance was found to affect both Emotions and Confidence subcategories. Higher subjective running ability and greater limb symmetry in the medial SLH were thus positively associated with each dimension of psychological readiness.

Several previous studies have reported on relationships between total ACL‐RSI score and physical function [13, 14]. However, only one study has analysed the relationship between subcategory scores and physical function [12]. In that study, relationships between Risk Appraisal score and anterior SLH distance laterality, visual analogue scale (VAS) score for limitation in sports, and Knee Osteoarthritis Outcome Score (KOOS) were analysed in patients at 2 years after reconstruction [12]. In the group unable to return to sports, Risk Appraisal score was strongly associated with the QOL subscore from the KOOS and the VAS score for limitation in sports, while SLH laterality showed no significant associations [12]. In the present study, anterior SLH distance LSI was related to risk appraisal. This difference in results may be mainly attributable to differences in the interval from reconstruction to testing and in the status of having made a return to sports of the subject. Lateral SLH distance LSI, which was not analysed in previous studies, was also associated with risk appraisal. Multiple regression analysis revealed that subjective running ability affected risk appraisal. These results suggest that 6 months after reconstruction, increasing the ability to run and SLH distances in both the anterior and lateral directions will lead to improved results in risk appraisals. On the other hand, a recent study in female patients 7 months after reconstruction reported that higher Risk Appraisal scores were associated with a second injury [10]. Younger age was associated with higher Risk Appraisal score [34]. From the perspective of preventing reinjury, it is controversial that a higher Risk Appraisal score is better for young female athletes.

No previous studies have analysed the relationships between emotions, confidence and physical functions. In the present study, we identified subjective running ability and LSI of medial SLH distance as important factors. These results suggest that increasing the ability to run and SLH in the medial direction will lead to improvements in both emotions and confidence. However, regarding Emotions scores, a previous study has reported that men have higher scores, so gender must be taken into account when interpreting the results [34].

The cross‐sectional design restricted the identification of causal relationships between physical function factors and ACL‐RSI scores. A small sample size tends to affect the results of regression analysis and the involved limb showed a large 95% CI for the coefficient. This suggests that the measure is unreliable and the position of the real value is uncertain. Therefore, the ability to interpret the contribution of the involved limb variable to the overall ACL‐RSI value remains limited. Subcategory scores are affected by age and gender, so these must be taken into account when generalizing the findings [10, 34]. In addition, the focus on hamstring tendon reconstruction limits the generalizability of findings to broader populations and different types of reconstructions.

Despite these limitations, no previous studies have provided details of physical function factors for ACL‐RSI subcategory scores from multiple categorical variables among patients seeking a return to sport at 6 months after surgery. A previous study comparing subcategory scores the day before reconstruction and at 6 months after surgery found that only the Risk Appraisal score showed a lack of significant improvement [11]. Even after 6 months postoperatively, Risk Appraisal scores were more difficult to improve than Emotions and Confidence scores, with particularly large differences in Risk Appraisal scores seen between a group that returned to sports 2 years after surgery and a group that did not [12]. Such findings suggest that trends in improvement for the three subcategory scores are not uniform, and that the physical functions associated with each also show some differences. The first 6 months following reconstruction represent an important period for rehabilitation to improve psychological readiness and physical function factors for a subsequent full return to sport [35, 36]. Clarifying the physical function factors of subcategory scores in addition to ACL‐RSI total score at 6 months after reconstruction is therefore important. The findings of this study could contribute insights into the nuanced relationship between specific physical function factors and the different dimensions of the ACL‐RSI scale. The emphasis on subjective running ability and SLH distances underscores the importance of targeted interventions for improving patient confidence, risk appraisal, and emotional well‐being during the return‐to‐sport phase.

## CONCLUSIONS

This study highlights the differential impact of physical function factors on specific subcategories of the ACL‐RSI scale, providing clinicians with insights for designing targeted rehabilitation strategies. This original paper suggests the importance of analysing factors related to subcategory scores in addition to total ACL‐RSI score, and could contribute to the understanding of determinants for a successful return to sport following ACL reconstruction.

## AUTHOR CONTRIBUTIONS

Jun‐ya Aizawa and Kenji Hirohata contributed substantially to the conception or design of the manuscript; Jun‐ya Aizawa, Shunsuke Ohji, Takehiro Ohmi, and Sho Mitomo contributed substantially to the acquisition, analysis, and interpretation of the data. All authors participated in drafting the manuscript. Hideyuki Koga and Kazuyoshi Yagishita revised the manuscript critically. All authors contributed equally to the manuscript and read and approved the final version of the manuscript.

## CONFLICT OF INTEREST STATEMENT

The authors declare no conflict of interest.

## ETHICS STATEMENT

The Investigation was performed at the Tokyo Medical and Dental University, Bunkyo‐ku, Tokyo, Japan. The institutional review board at our institution approved the study in accordance with the Declaration of Helsinki (approval no. M2019‐019). All participants provided written, informed consent prior to enrolment in this trial.

## Data Availability

The data sets used and/or analysed during the current study are available from the corresponding author on reasonable request.
